# Time series modeling of pneumonia admissions and its association with air pollution and climate variables in Chiang Mai Province, Thailand

**DOI:** 10.1007/s11356-018-3284-4

**Published:** 2018-09-26

**Authors:** Apaporn Ruchiraset, Kraichat Tantrakarnapa

**Affiliations:** Department of Social and Environmental Medicine, Faculty of Tropical Medicine, Mahidol University, Bangkok, Thailand

**Keywords:** Pneumonia, ARIMA, ARIMAX, PM10, Climate change, Air pollution, Respiratory disease

## Abstract

This study aimed to predict the number of pneumonia cases in Chiang Mai Province. An autoregressive integrated moving average (ARIMA) was used in data fitting and to predict future pneumonia cases monthly. Total pneumonia cases of 67,583 were recorded in Chiang Mai during 2003–2014 that the monthly pattern of case was similar every year. Monthly pneumonia cases were increased during February and September, which are the periods of winter and rainy season in Thailand and decreased during April to July (the period of summer season to early rainy season). Using available data on 12 years of pneumonia cases, air pollution, and climate in Chiang Mai, the optimum ARIMA model was investigated based on several conditions. Seasonal change was included in the models due to statistically strong season conditions. Twelve ARIMA model (ARMODEL1–ARMODEL12) scenarios were investigated. Results showed that the most appropriate model was ARIMA (1,0,2)(2,0,0)[12] with PM10 (ARMODEL5) exhibiting the lowest AIC of − 38.29. The predicted number of monthly pneumonia cases by using ARMODEL5 during January to March 2013 was 727, 707, and 658 cases, while the real number was 804, 868, and 783 cases, respectively. This finding indicated that PM_10_ held the most important role to predict monthly pneumonia cases in Chiang Mai, and the model was able to predict future pneumonia cases in Chiang Mai accurately.

## Introduction

The autoregressive integrated moving average (ARIMA) model was first proposed in 1976 and widely used for predicting and early warning analyzing of infectious diseases (Luz et al. 2008; Reichert et al. 2004; Yi et al. 2007) and for predicting future air quality status from various aspects of development in several countries (Konovalov et al. 2009; Pedro Muñoz Miguel et al. 2017). In addition, examining associations between environmental factors and adverse health outcomes is more advantages using the ARIMA model (Imai et al. 2015; Sharafi et al. 2017; Unkel et al. 2012). To improve forecasting performance, exogenous variables were included in the ARIMA model renamed the ARIMAX model.

Much evidence suggested that environmental air pollution, such as particulate matter (PM_10_), carbon monoxide (CO), nitrogen dioxide (NO_2_), sulfur dioxide (SO_2_), and ozone (O_3_), have adverse consequences for respiratory diseases (Luong et al. 2017; Phung et al. 2016; Shen et al. 2017; Zhu et al. 2017). Additionally, climatic variables (temperature, humidity, and rain) have also been reported by a number of studies to be associated with pneumonia hospitalization (Kim et al. 2016; Liu et al. 2014). Pneumonia is a serious infectious respiratory disease worldwide, especially among children. Of the estimated nine million child deaths in 2007, around 20% or 1.8 million were due to pneumonia (WHO and UNICEF 2009). To control this risk, the World Health Organization (WHO) and United Nations International Children’s Emergency Fund (UNICEF) launched a Global Action Plan for Pneumonia Prevention and Control (WHO and UNICEF 2009) in 2009. In Thailand, however, only few officials publish concerning pneumonia incidence. In Nakhon Phanom Province (northeast Thailand), pneumonia incidence was 831/100,000/year and 495/100,000/year in Sa Kaeo Province (east Thailand) from 2002 to 2003 (Jordan et al. 2009).

Therefore, this study aimed to first analyze the time series pattern of pneumonia administration in Thailand and obtain an appropriate model to predict future pneumonia cases using the ARIMA model. Since evidence suggests that air pollution and climate variables are important causes correlated with pneumonia incidence, a number of exogenous variables were included in the model. Chiang Mai Province, northern Thailand was chosen as a representative area because a high number of pneumonia admissions occur. Furthermore, this area usually faces air pollution during dry season when haze related to open burning occurs (Chantara et al. 2012) along with forest fires (Sillapapiromsuk et al. 2013).

## Method

### Study area

**Fig. 1:**
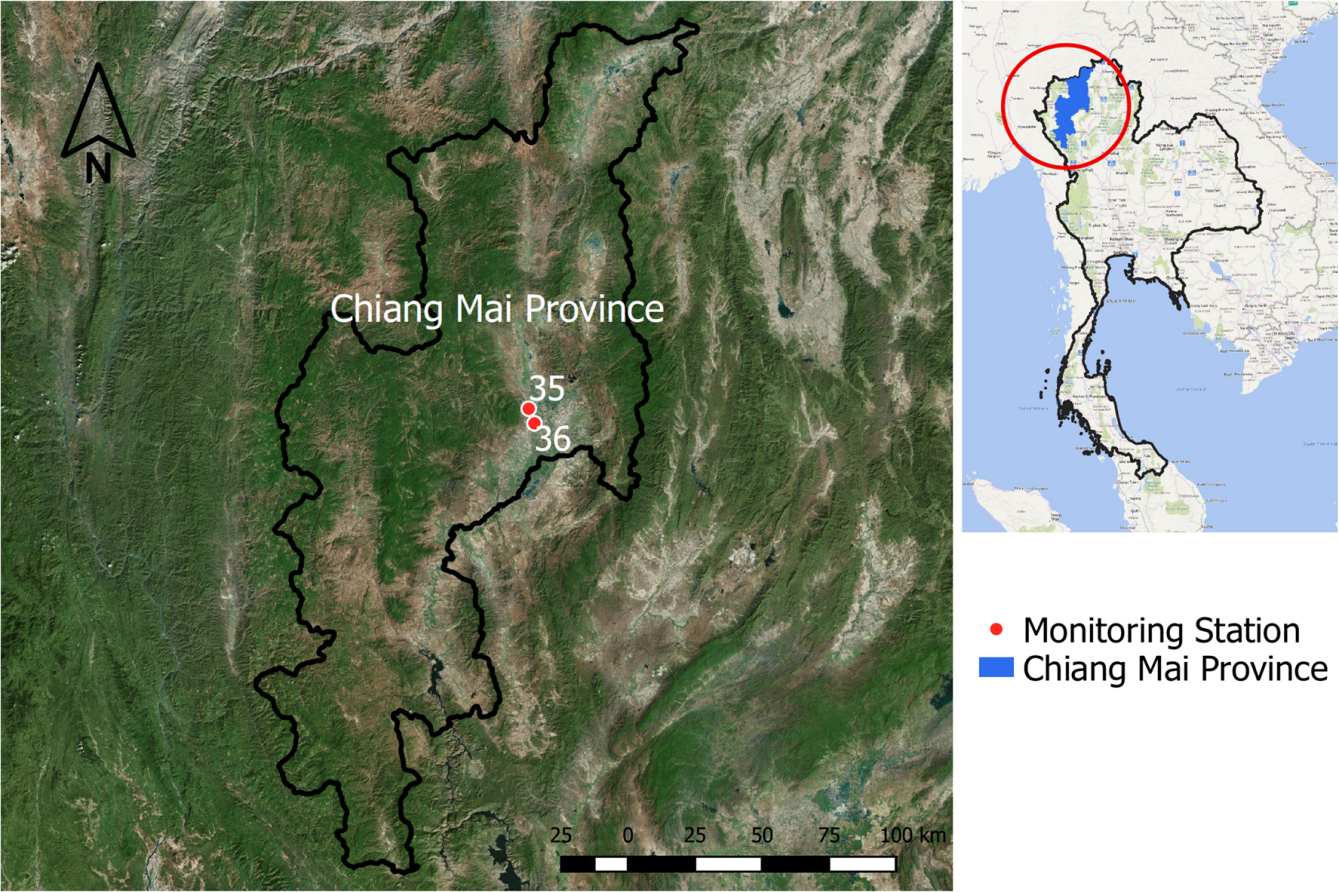
Chiang Mai, Thailand: the study area

Chiang Mai (Fig. 1), the second largest city in Thailand, covering an area of approximately 20,170 km^2^, is divided in 25 districts. The total population is 1,682,164 (30 June 2015) with a total of 742,489 households (Chiang Mai governor office 2018). The three seasons in Chiang Mai comprise winter (November to February), summer (March to May), and rainy season (June to October).

### Data sources and data management

Air pollution and climate data in Chiang Mai Province were acquired from the Pollution Control Department (PCD), Ministry of Natural Resources and Environment, Thailand. Two PCD monitoring stations located in Chiang Mai are in Mueang Chiang Mai District (station numbers 35 and 36), as presented in Fig. 1. Air pollution data consist of PM_10_, CO, NO_2_, SO_2_, and O_3_ and climate data comprise rain, humidity, and temperature. Both were normally documented on an hourly basis. All missing data were handled using mean substitution method and then hourly concentrations were averaged to give monthly concentrations.

Pneumonia case admissions in Chiang Mai were obtained from the Ministry of Public Health (MOPH), Thailand. Pneumonia cases were classified using code 31 according to one disease surveillance report (Report 506). Both pneumonia case admissions and air pollution data for Chiang Mai were obtained from 2003 to 2014. For the model prediction purpose, the data were divided in two groups. The first group covered 10 years (2003 to 2012), which was purposed for model fitting, and the remaining 2 years of data (2013 to 2014) was used for model prediction.

### ARIMA and ARIMAX model development

The model was developed using RStudio, Version 1.0.136. First, data were identified for a suitable model, and the stationarity of the time series was determined. To obtain a stationary time series, natural log transformation and differencing process were employed. After that, the stationary time series data was fit to the ARIMA (p, d, q)x(P, D, Q)s model where: p, d, and q represented autoregressive (AR) order, differencing order, and moving average (MA), respectively. Then, P, D, and Q referred to AR, differencing and MA terms for the seasonal part of the ARIMA model, where s represented the number of periods in each season. The general multiplicative seasonal ARIMA model used for this study can be written as Eq. (1), which more described detail was in Box et al. (2015).

1 $$ \upphi \left(\mathrm{B}\right){\Phi}_{\mathrm{P}}\left({\mathrm{B}}^{\mathrm{s}}\right){\nabla}^{\mathrm{d}}{\nabla}_{\mathrm{s}}^{\mathrm{D}}{\mathrm{z}}_{\mathrm{t}}={\uptheta}_{\mathrm{q}}\left(\mathrm{B}\right){\Theta}_{\mathrm{Q}}\left({\mathrm{B}}^{\mathrm{s}}\right){\mathrm{a}}_{\mathrm{t}} $$

where ϕ(B) and θ(B) are the polynomials in B degree of p and q, respectively. Φ_P_(B^s^) and Θ_Q_(B^s^) are the polynomials in B degree of P and Q, respectively. z_t_ is an observable time series. a_t_ is a white noise process.

By using auto.arima with stepwise function from the forecast package in R, we obtained the best order for the (p, d, q)x(P, D, Q) part of the model (ARMODEL1). After that, we simulated the ARIMA model with another 11 scenarios (as presented in Table 2). Exogenous variables were included in the model, named the ARIMAX model (ARMODEL2–ARMODEL12). In the ARIMAX model, we separated exogenous variables in two groups, air pollution variables (PM_10_, CO, NO_2_, SO_2_, O_3_) and climate variables (rain, humidity, and temperature). The ARMODEL2 comprised the ARIMA model with both air pollution and climate variables. ARMODEL3 and ARMODEL4 comprised the ARIMA model with five air pollution and three climate variables, respectively. Moreover, the individual exogenous variable was included in the ARIMA model separately (ARMODEL5 to ARMODEL12). Finally, we selected the best fitting model to predict future pneumonia cases in Chiang Mai according to the Akaike Information Criterion (AIC) value.

## Results

### Explanatory statistics

A summary of data statistics is presented in Table 1. The number of pneumonia case admissions in both hospitals and primary health care units in Chiang Mai from January 2003 to December 2014 totaled 67,583 cases. Maximum pneumonia cases were 978 cases/month, while the minimum pneumonia cases were 132 cases/month. Monthly average concentration of PM_10_, CO, NO_2_, SO_2_, and O_3_, obtained from averaged values of two PCD’s monitoring stations located in Chiang Mai, was in the range of 18.5 to 189.4 μg/m^3^, 0.16 to 1.79 ppm, 6.02 to 30.51 ppb, 0.21 to 3.10 ppb, and 7.31 to 40.57 ppb, respectively. The three climatic factors considered in this study, i.e., rain, humidity, and temperature ranged from 0.00 to 0.61 mm, 43.45 to 88.99 %RH, and 20.1 to 31.58 °C, respectively.

**Table 1:** Descriptive statistics of data

Variable	Range	Mean ± s.d.
Monthly pneumonia cases	132–978	496 ± 179
Air pollution data (monthly average)		
PM_10_(μg/m^3^)	18.5–189.4	48.0 ± 30.2
CO (ppm)	0.16–1.79	0.63 ± 0.23
NO_2_ (ppb)	6.02–30.51	13.01 ± 5.58
SO_2_ (ppb)	0.21–3.10	1.04 ± 0.50
O_3_ (ppb)	7.31–40.57	19.67 ± 7.79
Climate data (monthly average)		
Rain (mm.)	0.00–0.61	0.13 ± 0.14
Humidity (%RH)	43.45–88.99	69.20 ± 10.70
Temperature (°C)	20.1–31.58	26.8 ± 2.3

**Fig. 2:**
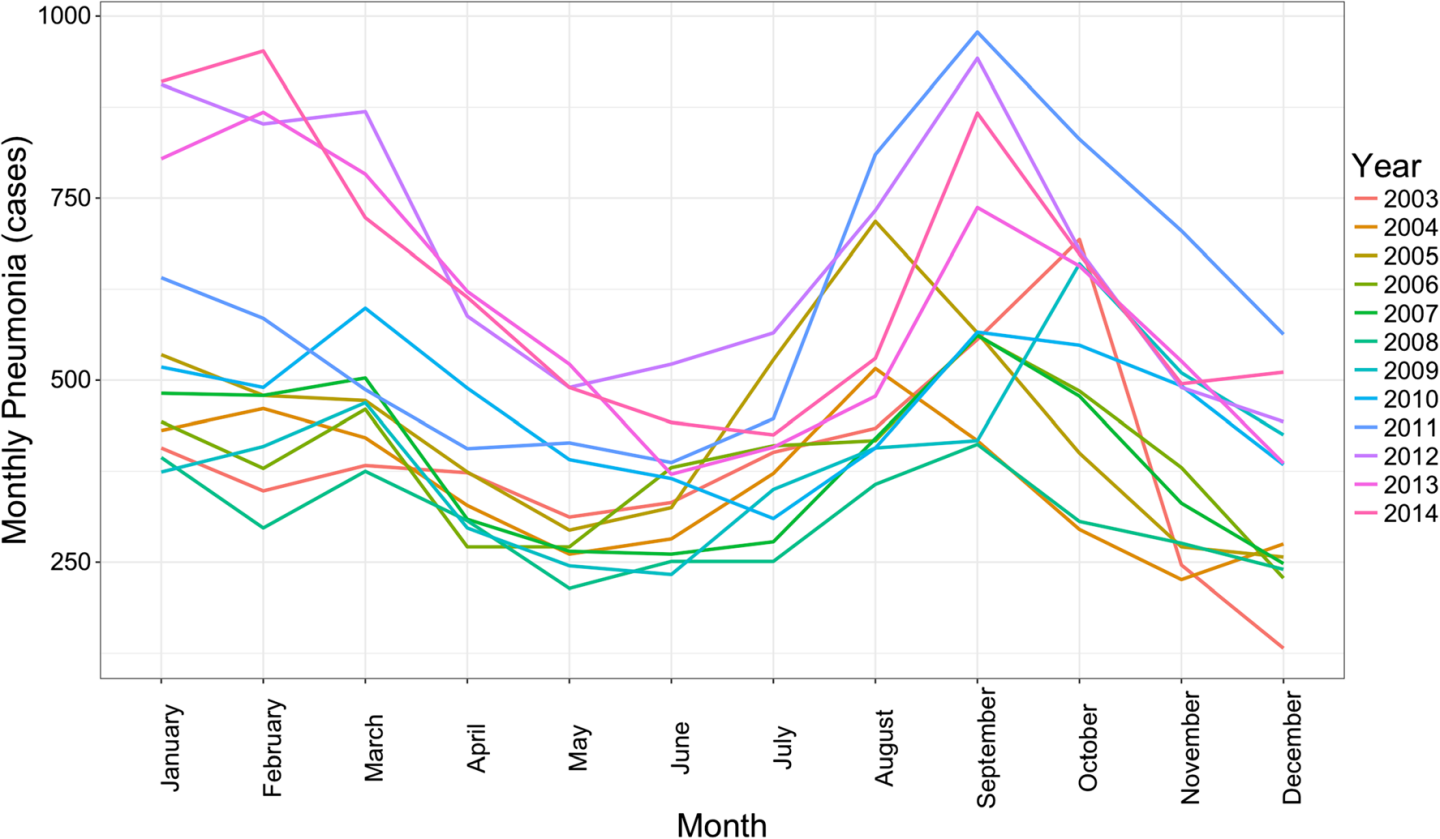
Time series plot of monthly pneumonia case in Chiang Mai from 2003 to 2014

**Fig. 3:**
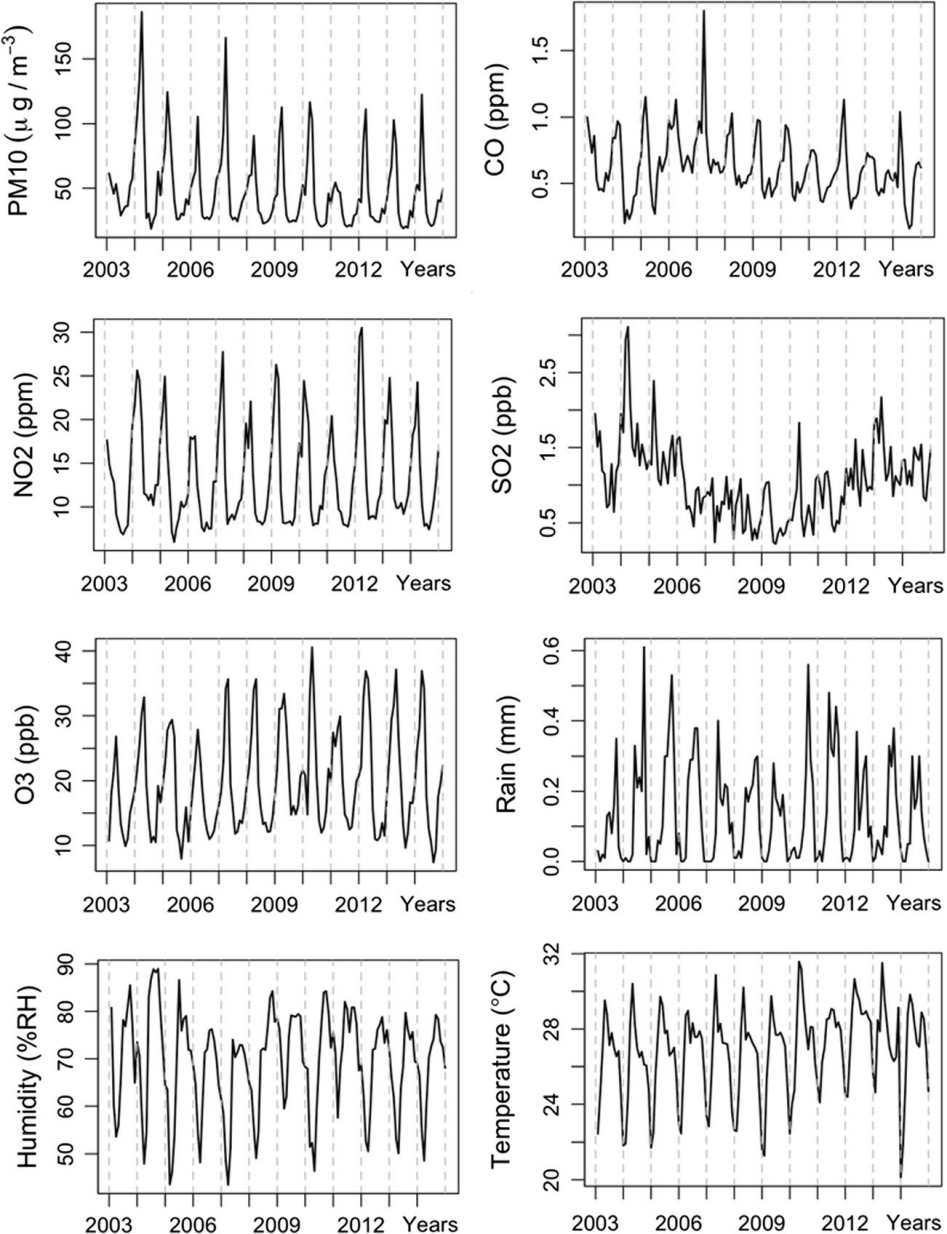
Time series plot of monthly air pollution variables and climate variables from 2003 to 2014 in Chiang Mai

Time series plot of monthly pneumonia cases in Chiang Mai during the period of this study is illustrated in Fig. 2. Monthly pneumonia cases were increased in January and gradually decreased afterward to the summer period of May. After the summer period, the pneumonia cases were increased again from July to reach the peak point in September. It was found that the monthly pneumonia cases were increased during the last 3 years of the study (2012–2014) as presented in the purple, pink, and dark pink line. Monthly average concentration of air pollution and climate variable was presented in Fig. 3. PM_10_, CO, NO_2_, and O_3_ concentrations were similar patterns during the study period. However, SO_2_ concentration show high concentration during 2003 to 2005 and decreased after 2006 due to the regulation of fuel type intervention and it was increased again after 2011. Rain and humidity trend were gradually decreased while temperature was gradually increased.

### ARIMA model

**Fig. 4:**
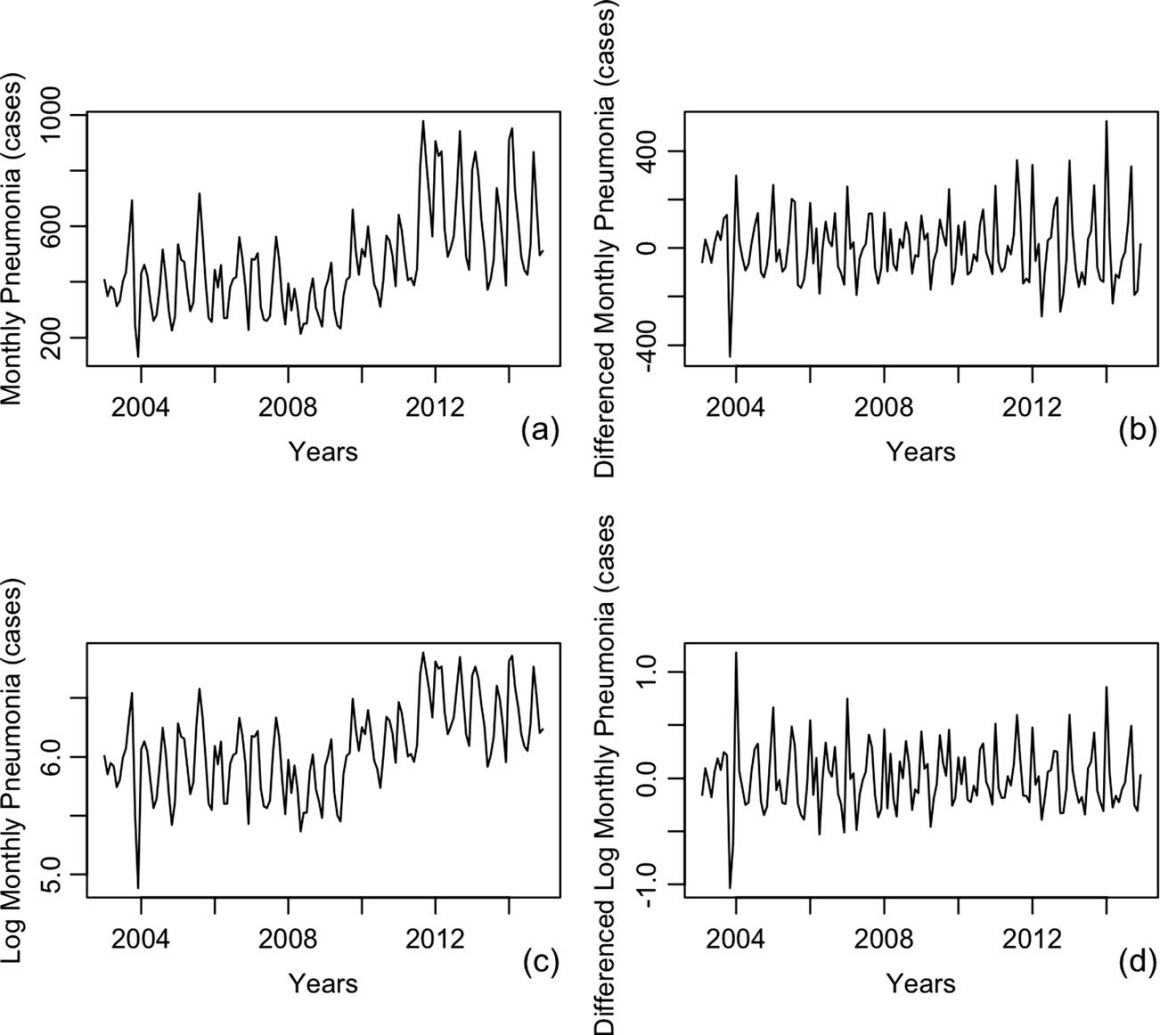
Transforming data to make data stationary

ARIMA modeling began by estimating the degree of autoregressive (p), differencing (d), and MA (q). However, we needed to check the time series before starting the modeling process. Using an augmented Dickey-Fuller test (ADF), we found our data was not stationary. To fix this problem, natural log transformation and differencing were performed to make the data stationary. Figure 4a, b, c presents monthly plots of pneumonia cases, differencing of monthly pneumonia cases and natural log transformed monthly pneumonia cases, respectively. The plot of Fig. 4a shows that mean monthly pneumonia case is a function of time (data are nonstationary). To make the dataset stationary, we tried differencing and making a natural log transformation in the data series. Figure 4a, b, c shows the data series achieved satisfying conditions. Moreover, we endeavored to use both differencing and natural log transformation in the data series resulting in a more stationary series (Fig. 4d). Subsequently, differencing and natural log transformation of the data series were implemented before ARIMA modeling processes.

**Fig. 5:**
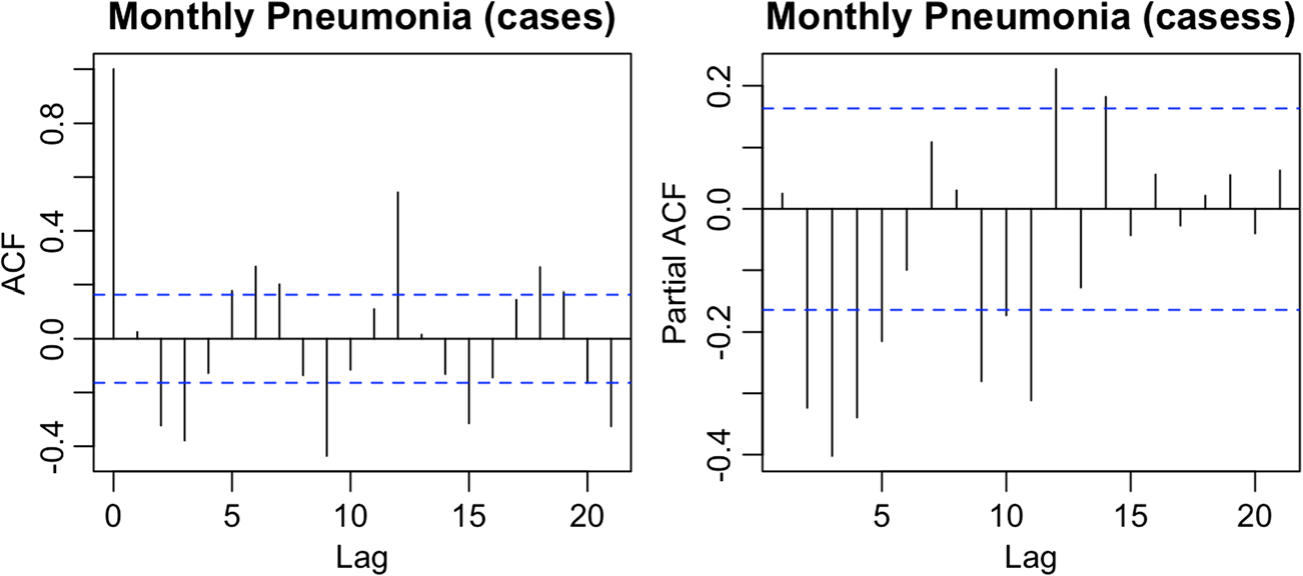
ACF and PACF of the first difference order of log of pneumonia cases in Chiang Mai

Seasonality was also considered in the study, as presented in Fig. 5. The ACF (autocorrelation function) plot of seasonality exhibited strong positive autocorrelations at any time lag 6, 12, and 18 suggesting a strong seasonal component. Hence, the seasonal component was considered for the ARIMA models in this study.

To examine the best order for the ARIMA model (p, d, q and P, D, Q), we used the auto.arima function in the forecast package attached with stepwise function to identify the best order. Stepwise function selected the best order of the ARIMA model based on AIC value. To explore the optimum ARIMA model, we used 12 different ARIMA models with various scenarios as presented in Table 2. First of all, we examined the ARIMA model (without exogenous variable) named ARMODEL1. The result from stepwise function indicated that the best order of the ARMODEL1 was ARIMA (1,0,2)(2,0,0)[12] (AIC − 30.77). We hypothesized that exogenous variables, air pollution, and climate variables would have an association with monthly pneumonia cases. ARMODEL2 to ARMODEL12 were examined to identify which exogenous variables should be included in the model.

**Table 2:** Model scenario in this study

Model	Exogenous variables							
	Air pollution					Climate		
	PM_10_	CO	NO_2_	SO_2_	O_3_	Humidity	Rain	Temperature
ARMODEL1								
ARMODEL2	✘	✘	✘	✘	✘	✘	✘	✘
ARMODEL3	✘	✘	✘	✘	✘			
ARMODEL4						✘	✘	✘
ARMODEL5	✘							
ARMODEL6		✘						
ARMODEL7			✘					
ARMODEL8				✘				
ARMODEL9					✘			
ARMODEL10						✘		
ARMODEL11							✘	
ARMODEL12								✘

✘ Exogenous variable(s) included in the model.

The best model based on the lowest AIC value of each scenario is shown in Table 3. The most appropriate ARMODEL1 was ARIMA(1,0,2)(2,0,0)[12] for which the lowest AIC was − 30.77. The most appropriate order of ARIMA models with exogenous variables for ARMODEL2, ARMODEL3, and ARMODEL4 were ARIMA(1,0,2)(2,0,0)[12] (AIC − 29.83); ARIMA(1,0,2)(2,0,0)[12] (AIC − 32.60); ARIMA(1,0,2)(2,0,0)[12] (AIC − 34.08), respectively. In this point of view, the ARMODEL4 exhibited the lowest AIC of − 34.08, which implied that climate variables had more impact on monthly pneumonia cases than air pollution variables.

**Table 3:** Summary of ARIMA and ARIMAX model prediction of monthly pneumonia cases in Chiang Mai Province

Model		ARMODEL1 ARIMA(1,0,2)(2,0,0)[12]	ARMODEL2 ARIMA(1,0,2)(2,0,0)[12] With Pollution and Climate variables	ARMODEL3 ARIMA(1,0,2)(2,0,0)[12] With Pollution variables	ARMODEL4 ARIMA(1,0,2)(2,0,0)[12] With Climate variables	ARMODEL5 ARIMA(1,0,2)(2,0,0)[12] With PM_10_	ARMODEL6 ARIMA(1,0,2)(2,0,0)[12] With CO	ARMODEL7 ARIMA(1,0,2)(2,0,0)[12] With NO_2_	ARMODEL8 ARIMA(1,0,2)(2,0,0)[12] With SO_2_	ARMODEL9 ARIMA(1,0,2)(2,0,0)[12] With O_3_	ARMODEL10 ARIMA(1,0,2)(2,0,0)[12] With Humidity	ARMODEL11 ARIMA(1,0,2)(2,0,0)[12] With Rain	ARMODEL12 ARIMA(1,0,2)(2,0,0)[12] With Temperature
Fit	RMSE	0.19	0.18	0.18	0.18	0.18	0.19	0.18	0.19	0.19	0.19	0.18	0.19
	AIC	− 30.77	− 29.83	− 32.60	− 34.08	− 38.29	− 33.90	− 35.53	− 28.81	− 30.29	− 30.43	− 37.98	− 30.17
	AICc	− 29.77	− 25.22	− 29.69	− 32.06	− 36.99	− 32.60	− 34.23	− 27.51	− 28.99	− 29.14	− 36.69	− 28.87
	BIC	− 11.26	11.98	− 0.85	− 6.2	− 15.99	− 11.6	− 13.23	− 6.51	− 7.99	− 8.13	− 15.68	− 7.87
AR1	Coef.	0.94***	0.95***	0.95***	0.95***	0.94***	0.95***	0.93***	0.94***	0.94***	0.95***	0.94***	0.94***
MA1	Coef.	− 0.20*	− 0.17	− 0.13	− 0.25**	− 0.14	− 0.17	− 0.12	− 0.21*	− 0.21*	− 0.24**	− 0.21*	− 0.24*
MA2	Coef.	− 0.46***	− 0.55***	− 0.56***	− 0.48***	− 0.53***	− 0.53***	− 0.52***	− 0.47***	− 0.47***	− 0.49***	− 0.47***	− 0.45***
SAR1	Coef.	0.40***	0.44***	0.42***	0.45***	0.42***	0.40***	0.42***	0.40***	0.41***	0.46***	0.41***	0.40***
SAR2	Coef.	0.39***	0.38***	0.39***	0.36***	0.39***	0.39***	0.39***	0.39***	0.38***	0.37***	0.39***	0.39***
PM10	Coef.		0.00	0.00		0.00**							
CO	Coef.		0.11	0.09			0.25*						
NO_2_	Coef.		0.00	0.00				0.02**					
SO_2_	Coef.		− 0.03	− 0.05					0.01				
O_3_	Coef.		− 0.00	− 0.00						0.01			
Humi	Coef.		− 0.01		− 0.01**						− 0.01**		
Rain	Coef.		− 0.02		0.02							− 0.19	
Temp	Coef.		0.01		0.01								0.03

Significant codes: *** *p* < 0.001, ** *p* < 0.01, * *p* < 0.05

RMSE = root mean square error; AIC/AICc = Akaike information criterion; BIC = Bayesian information criterion; AR1 = the first-order autoregressive process; MA1 = the first-order moving average; MA2 = a second-order moving average; SAR1 = the first-order seasonal difference

**Fig. 6:**
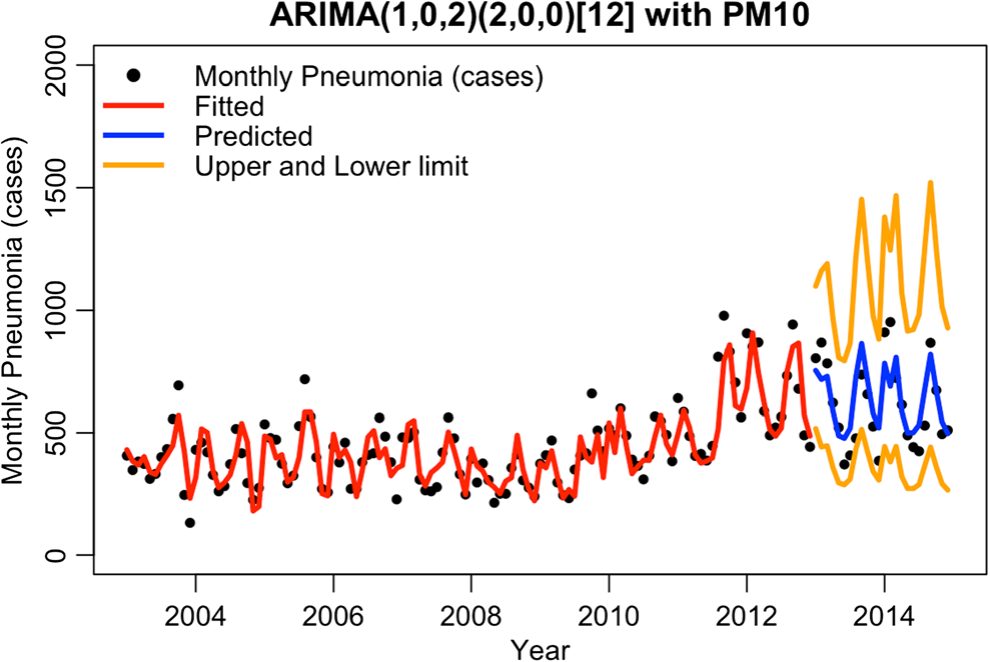
Fitted and predicted value of ARMODEL5 in Chiang Mai Province

Models with individual exogenous variables (ARMODEL5 to ARMODEL12) were investigated to determine, which exogenous variable showed significant associations with monthly pneumonia cases. As shown in Table 3, ARIMA(1,0,2)(2,0,0)[12] with PM_10_ (ARMODEL5) had the lowest AIC of − 38.29 and was the best model in this study because it exhibited the lowest AIC value. Figure 6 presents the fitted and predicted value of ARMODEL5. The black dots in Fig. 6 represent actual pneumonia cases monthly. The red line represents the fitted value from ARMODEL5 from 2003 to 2012. The blue line shows the predicted value using the model from 2013 to 2014, while upper and lower limits (defined by two times standard deviation) are displayed with orange lines on either side of predicted line. The real number of monthly pneumonia cases was 407, 408, and 383 cases in January to March 2003, while the fitted by using ARMODEL5 was 431, 384, and 368 cases, respectively. The predicted number of monthly pneumonia cases by using ARMODEL5 during January to March 2013 was 727 (517–1099), 707 (443–1162), and 658 (447–1190) cases (values in the bracket were lower and upper limit), while the real number was 804, 868, and 783 cases, respectively. The results indicated the predicted numbers were close to the real situation.

## Discussion

This study aimed to predict monthly pneumonia cases in Chiang Mai by applying ARIMA and ARIMAX modeling. Pattern of pneumonia in Chiang Mai during study period was similar pattern in each year that showed the increasing number was observed during Februry and September, which are classified as the periods of winter and rainy season in Thailand and it was decreased during April to July (the period of summer season to early rainy season).

When comparing between air pollution (ARMODEL3) and climate variables (ARMODEL4), the results in this study indicated that climate variables played a more important role concerning pneumonia than air pollution variables because ARMODEL4 exhibited lower AIC values than ARMODEL3. However, worldwide studies have demonstrated that both air pollution and climate variables affect pneumonia (Davis et al. 2016a; Kim et al. 2016; Paynter et al. 2013; Xu et al. 2014). Several research studies have focused on the relation between pneumonia admissions and air pollution. Chiu et al. (2009) studied the association of air pollutants and hospital admissions for pneumonia in Taipei, Taiwan. They reported that increased ambient of air pollutants (PM_10_, CO, and O_3_) increased the risk of hospital admissions for pneumonia. The study in Thailand by Guo et al. (2014) indicated that PM_10_ was significantly related to respiratory mortality. When focusing on air pollution variables (PM_10_, CO, NO_2_, SO_2_, O_3_), PM_10_ exhibited the most robust association with monthly pneumonia cases because the ARMODEL5 presented the lowest AIC values when compared with the others. PM_10_ concentration indicated the positive association with monthly pneumonia admission in Chiang Mai Province; more PM_10_ concentration was also increased in number of monthly pneumonia cases particularly the early period of each year. Particulate matter (PM) has been found to be the most common and consistent variable associated with pneumonia (Janssen et al. 2013; Zanobetti and Schwartz 2006). Increased levels of PM were associated with increased levels of respiratory disease among children and older adults (Wang et al. 2015). Medina-Ramon et al. (2006) stated that increased PM_10_ during the warm season resulted in increased pneumonia admissions at lag 0. Mechanisms of PM_10_ on adverse respiratory effects remain unclear (Medina-Ramon et al. 2006). However, a probable reason suggested by Xing et al. (2016) was PM_2.5_ (particles less than 2.5 μm in diameter) can penetrate deeply in the lung, irritate, and corrode the alveolar wall and consequently impair lung function. Even though this study used PM_10_, PM_2.5_ is the subset of PM_10_.

When focusing on climate variables (humidity, rain, and temperature), rain was seen to play the most important role with pneumonia admissions in Chiang Mai. Selecting AIC values, ARMODEL11 exhibited the lowest AIC of − 37.98 but its coefficient (see Table 3) was insignificant. Therefore, ARMODEL10 (ARIMA model with humidity, AIC of − 30.43) was chosen when considering only climate variables. Association of humidity and pneumonia has been reported since 1980 (Bull 1980). Bull (1980) stated that changes in temperature and humidity were highly and significantly correlated with changes in death rate from pneumonia exhibiting a positive correlation of high humidity and temperature. Moreover, Davis et al. (2016b) stated that several research studies indicated that humidity was one of the most highly associated variable with pneumonia. The study in New Zealand by Davis et al. (2016a) found that respiratory infection was enhanced during unusually cold conditions and during conditions with unusually low humidity.

Although the coefficient of rain in ARMODEL11 was not significant, it exhibited the lowest AIC value among these groups. Consequently, we decided to use ARMODEL11 to predict future monthly pneumonia cases. Evidence supporting this decision has been shown by several research studies. Singh et al. (2014) studied the association of rain-wetting with the occurrence of pneumonia during an outbreak of influenza A (H1N1) pdm09 virus infection. They found that the number of pneumonia patients increased during periods of greater rainfall and rain-wetting and may be an important risk factor for the occurrence of pneumonia. Nevertheless, different results were shown by the Hei Collaborative Working Group on Air Pollution et al. (2012) who found a relation between pneumonia hospitalizations and levels of PM_10_, SO_2_, and NO_2_ between the months of November and April annually (data of 3 years in Vietnam) but found no association in the rainy season months of May through October. However, several limitations were reported in this study, such as the lack including age and sex and the lack time to be reconsidered in a future study. Moreover, it should be noted that air pollution and climate variables used in this study were derived from two monitoring stations located in Chiang Mai Province.

## Conclusion

The study aimed to predict the number of pneumonia cases in Chiang Mai Province. Twelve years of monthly pneumonia cases, air pollution, and climate variables in Chiang Mai was used to investigate the optimum of model with several scenarios. Seasonality was included in this model because it showed a strong and significant association. ARMODEL5 (ARIMA(1,0,2)(2,0,0)[12] with PM_10_) was the most appropriate model based on current data. This suggested that PM10 was the most important factor to predict monthly pneumonia cases in Chiang Mai.
